# Letter from London

**Published:** 1882-01

**Authors:** W. T. Belfield

**Affiliations:** London


### Foreign Correspondence.

### ARTICLE VII.

LONDON, Dec. 3, 1881.

EDITORS CHICAGO MEDICAL JOURNAL AND EXAMINER:—Each of the fourteen general hospitals has of this city has its "out-patient" department; a dispensary in fact. The attendance is enormous, and divided into sections according to the nature of their ailments, these patients furnishing abundant facilities for medical instruction. Every department of therapeutics is represented at each hospital by a specialist, and here is laid the foundation for that broad knowledge of and practical acquaintance with the entire range of medicine and surgery which distinguishes the young English graduate. Every student serves as dresser or assistant in each of the various departments; with the surgeon he has opportunities for dressing, with the gynæcologist, for using the speculum, with the ophthalmic surgeon for employing the ophthalmoscope, and so on throughout the whole list. Certificates of such service are required before the student is admitted to the privileges of study in the wards. Here he becomes clinical clerk to a physician or dresser to a surgeon, and thus comes into intimate contact with the severe cases, medical and surgical.

In his four years of study, therefore, he has—he is *compelled* to have — vast opportunities, probably unequalled outside of English schools, for immediate and practical observation of disease. These advantages can be realized when one reads that the privileges of the London Hospital, containing 800 beds, where were treated, during 1879, 6,361 in-patients and 47,998 out-patients (excluding 22,737 cases of diarrhœa), are divided among about one hundred students. The student revels, in fact, in a luxury of disease; but there is, unfortunately, one signal defect in the system, which vitiates the whole—the lack of instruction. I do not refer to didactic lectures, which in these days of good text-books can only exceptionally be regarded, except by a stretch of courtesy, as instruction; the London student is lectured enough, but he receives very little personal instruction in the examination of patients, in the use of the various means of diagnosis which the physician must employ. He may watch his teachers in their examinations; he is at liberty to examine patients himself; but he is not supervised, questioned, corrected, directed as he might and should be. Hence it is that at the end of his four years' work, after examining hundreds of cases, he is often unable to distinguish the crepitant râle of pneumonia from the sonorous râle of asthma; he regards as a granular cast a harmless collection of amorphous urates; he considers an opacity of the lens as a retinal hæmorrhage. These are not fancy pictures, but instances of mistakes "taken fron life." Yet so far as he can learn by observation and practice, without individual teaching, the London student is a model, and he is probably, on the whole, the best-informed man, for his years, in practical medicine and surgery, that I have as yet observed.

After securing his license to practice, or becoming "qualified," in local parlance, from the College of Physicians, or from that of Surgeons, or from any other of the nineteen corporations empowered to confer degrees in Great Britain, several professional paths are open to the youthful Esculapian, whose destructive tendencies are indicated not by a simple "M.D.," as is the case in every other civilized country, but by M.R.C.S., or L.R.C.P., or some equally imposing literary array. (I have counted no less than fifteen such capitals in procession after one name). He may become a general practitioner, or a consulting physician or surgeon. In the former case he displays, not simply a "shingle," but also a very suggestive, sanguinary looking red glass case, enclosing a gas jet, whose lurid glare notifies the world that a "Surgeon, etc." is domiciled there. I was at first curious to know the significance of that "etc.," until I one day noticed that one man unblushingly labeled himself "Surgeon and Accoucheur." It then dawned upon me that the "etc." was a euphemism, employed out of deference to the well-known delicacy (sic) of the British public. This "surgeon, etc." treats everything that falls into his net, receiving fees as low as a shilling, dispenses his own medicine, and sends for a "consultant" in any doubtful case. If he be very successful, he may in the course of time abandon general practice and become a consulting physician—the exception, however.

The true "consultant" has never sullied his reputation with general practice. After receiving his degree he becomes house-surgeon or physician somewhere; subsequently, if very "swell," he spends a few months "abroad, you know, aw;" then he opens a "consulting-room," usually in the East End; his consultations during the first few years being chiefly with his laundress and boon companions. Meanwhile he secures one of the numerous subordinate posts at a hospital, with a small salary—eighty to one hundred pounds—attached—as assistant demonstrator of anatomy, or clinical assistant, or registrar. With this income and his private resources he maintains his consulting-room and a supply of silk hats—both absolutely essential to professional dignity—and spends his spare time in study at special hospitals, and in reading for his fellowship examination. After a longer or shorter period he gets an appointment as assistant physician or surgeon, picks up two or three other appointments at various hospitals, and if he can subsist five or ten years longer, begins to get some private (consultation) practice. In another ten years he moves to the West End, procures a carriage and pair, with liveried coachman, and is a great man. I am acquainted with a gentleman who is assistant surgeon to a large general hospital, surgeon to an eye hospital, a skin hospital and a children's hospital; he has an elegant suite of rooms, and hopes some day to have a practice. In this way the Jonathan Hutchinsons are made—by devoting the first few (sometimes many) years of professional life to hospital work exclusively, renouncing all pecuniary considerations for the sake of a vast clinical experience.

At the various special hospitals, sixty-six in number (returns of new hospitals for last week not yet received), one sees enormous numbers of patients—each physician usually seeing from 125 to 250 during the afternoon. These "hospitals" are in fact but little more than dispensaries, since they have in many cases only ten to thirty beds each; the buildings are usually dwelling-houses, quite devoid of the necessary sanitary appointments. Many have originated in a "want long felt" by certain medical men, of opportunities for studying disease, opportunities which they were unable to obtain at regular hospitals. As a natural consequence, there has arisen a shameless abuse of "charity" by people abundantly able to pay for professional advice. In fact, the competition for patients is not limited to the small hospitals; a few days ago there appeared in one of the leading dailies a letter from a "grateful patient," extolling the care and skillful treatment which she had received at a certain large general hospital, and advising people who needed attendance to patronize that institution, "especially if they were able to pay eight shillings a day." A writer in a medical journal, commenting thereon, prophesies the appearance at no distant date of placards on the omnibuses and walls—"Try St. Thomas' Hospital; bones skillfully set; operation bloodless and painless; only eight shillings per day." "Go to Guy's Hospital for Mary Jane's champion poultices—two shillings," etc. There appear at intervals in the daily press articles from medical men urging the necessity for reforming and regulating the numerous medical charities of the city, but no one seems disposed to initiate the movement.

Medical practice in London presents few novel features, aside from its extreme conservatism. The surgeons use for fractures of the leg the wooden box with bran bags, and starch or gum bandages; for fractures of the thigh a weight and pulley and perineal band; they reduce downward dislocations of the shoulder by means of the heel in the axilla. They are adopting the Vienna treatment of ulcers, burns, fungous joint surfaces, etc., by iodoform in powder or solution. Mr. Lister and some others cover ulcers with iodoform, lay over this a piece of "protective," and cover this again with boracic lint—*i. e.*, lint which has been dipped in a saturated solution of boracic acid, crystals of which are deposited on the lint in drying. Another Vienna notion, Leiter's leaden tubes for conducting water of a constant temperature over an inflamed part, have also been introduced.

The physicians are remarkable, first, for their faith in remedies, and second, for the minuteness of detail with which they examine patients. They are not content with the stethoscope, thermometer and test tube; they examine the eye, including the fundus; the mouth and larynx: palpate the abdomen; hammer out the knee-jerks and all the other jerks; estimate the quantity of urea; count the blood-corpuscles; note patient's weight; test his sensation, general and special; secure tracings with the sphygmograph and cardiograph—not, of course, in every case, but certainly upon slight provocation. Nor is that all. Dr. Sansom, instigated by a Frenchman during the late Congress, undertakes to mark out by percussion the walls of the various cavities and the position of the several valves in the heart. He employs a pleximeter of ebonite, a simple cylinder with the diameter of a thin lead-pencil some two inches long, solid, with a flange surrounding either end, the upper one, for the percussing finger, about $\frac{5}{8}$, the other $\frac{3}{8}$ inch in diameter.

Mr. Spencer Wells recently removed a pregnant (six months) uterus, with the ovaries, for epithelioma of the cervix—the first case on record. Patient recovered from the operation—whether from the disease or not remains to be seen. Billroth performed (but did not record) a similar operation several months ago. His patient recovered also, but soon died from recurrence of the disease. Mr. Wells, in presenting a report of the case to the Medico Chirurgical Society, expressed his belief that radical measures were usually necessary in cancer of the cervix; that destruction of tissue by knife and caustic, after Marion Sims, rarely offered a probability of success.

Mr. Reeves has been discussing the relative merits of gastrostomy and œsophagostomy in stricture of œsophagus (particularly cancerous) not dilatable. He urges the opening of the œsophagus, unless stricture is very low, as more practicable and less dangerous. Unfortunately, his opinion does not, as yet, rest upon clinical experience, and is not very warmly endorsed by his brother surgeons. His operation for genu valgum, however, by chiselling away the condyle, and opening the joint, is recommended by several surgeons of experience.

Mr. Lister and his colleagues have been for some time treating transverse fractures of the patella by cutting down upon the bone and passing silver wires through holes bored through the separate fragments, the ends of the wires being then twisted together so as to hold the fractured surfaces in close apposition. It was claimed that the operation was (under the spray, of course) without danger, and secured the firmest possible union. I say *was* claimed; for since, in one instance, the knee-joint suppurated, we have heard less of the immunity of the operation.

At Moorfields Eye Hospital, the rage just now is "retinoscopy." With the mirror (about 9-in. focus) held forty-eight inches in front of the eye, light is thrown in at the usual angle; the mirror is then rotated on its vertical axis; if a shadow encroach on the illuminated pupil on the same side as the advancing edge of the mirror, the eye is myopic more than one diopter; if shadow appears on the opposite side, the eye is hypermetropic, emmetropic or myopic less than one diopter. Further examination reveals details which I won't inflict upon you; the method is certainly convenient and efficient for detecting certain anomalies of refraction, though it furnishes no information which can not be acquired by the usual methods.

The Listerian precautions are by no means universally practised here. In King's College Hospital, it is true, all the general surgeons follow the example of their chief; in St. Bartholomew's, on the other hand, better results are obtained by the "septic" method. In the Samaritan Hospital for Women, Mr. Thornton performs an ovariotomy one day with all the paraphernalia so fatal to bacteria; the next day Dr. Bantock makes an ovariotomy with scarcely a "smell" of carbolic acid. Between the two extremes may be observed several grades of caution; some omit the spray; others use it as a matter of form, and if at the end of the operation it is discovered that the carbolic acid bottle is empty, and that the spray has been simply steam, or if the perverse machine suddenly stops in the midst of the performance, and obstinately refuses to go, nobody feels very bad about it; it's a "good joke on Lister"—nothing more. The bacterial hallucination sometimes presents amusing phases. Not long ago a rigid apostle of Listerism made an amputation at the hip. The dressing was applied over the same regions as the gauze of a ballet-dancer. Whenever the patient desired to evacuate his bladder (fortunately it was a "he") the house-surgeon or dresser was summoned; the spray was directed so as to shower the patient's torrid zone; the dressing was carefully loosened, the necessary organ was fished up out of the depths and directed over a vessel, and then the gentle gurgle of the escaping renal secretion mingled sweetly with the murmured admiration of the bystanders, the fizz of the faithful spray, and (presumably) the teeth-gnashing of baffled bacteria. Needless to say that the patient died. W. T. BELFIELD.

---

CAPSICUM ANNUUM IN METRORRHAGIA.—M. Cheron in the *Revue Med. Chir. des Mal. des Femmes*, recommends strongly cayenne pepper in all forms of uterine hæmorrhage, whether due to fibroid tumors or to fungous endometritis, or even to epithelioma. He was first led to essay this remedy by the good effects observed from its use in hæmorrhoids. A large number of physiological experiments led him to consider it as having a special action on the vascular system, and hence on organs very rich in blood vessels, such as the utero-ovarian system, the organs of respiration and the brain.

Cayenne pepper acts like ergot on the non-striated muscular fibers of the vessels, either directly or through the vasomotor system, and it has the great advantage over ergot that it is well supported by the stomach, acting as a stimulant to its functions. The medicament can be given in pill form, two grains before each meal, increasing to four grains, or the watery extract in the same dose, or in tincture, much diluted and given more frequently.—*Med. and Surg. Reporter.*

